# Pre-notification and reminder SMS text messages with behaviourally informed invitation letters to improve uptake of NHS Health Checks: a factorial randomised controlled trial

**DOI:** 10.1186/s12889-019-7476-8

**Published:** 2019-08-22

**Authors:** Anna Sallis, Joseph Sherlock, Annabelle Bonus, Ayoub Saei, Natalie Gold, Ivo Vlaev, Tim Chadborn

**Affiliations:** 1 PHE Behavioural Insights, Public Health England, 6th Floor, Wellington House, 133-155 Waterloo Road, London, SE1 8UG UK; 2 Center for Advanced Hindsight, Social Science Research Institute, Duke University, 334 Blackwell Street, Suite 320, Durham, North Carolina 27701 USA; 3 HMRC, 100 Parliament Street, London, SW1A 2BQ England; 4 Ofgem, 10 South Colonnade, Canary Wharf, London, E14 4PU UK; 5 PHE Statistics, Modelling and Economics Department, Public Health England, Colindale Avenue Site, 61 Colindale Avenue, London, NW9 5EQ UK; 6 Faculty of Philosophy, Radcliffe Observatory Quarter 555, Woodstock Road, Oxford, OX2 6GG England; 7 Warwick Business School, University of Warwick, Coventry, CV4 7AL UK

**Keywords:** Behavioural insights, Cardiovascular disease, Invitation letters, NHS Health Checks, Pre-notifications, SMS, Reminders, Social norms, Planning prompts, Uptake, Text messages

## Abstract

**Background:**

The NHS Health Check (NHS HC) is a cardiovascular risk assessment to prevent cardiovascular disease. Public Health England (PHE) wants to increase uptake.

**Methods:**

We explored the impact of behaviourally informed invitation letters and pre-notification and reminder SMS on uptake of NHS HCs. Patients at 28 General Practices in the London Borough of Southwark who were eligible to receive an NHS HC between 1st November 2013 and 31st December 2014 were included. A double-blind randomised controlled trial with a mixed 2 (pre-notification SMS – yes or no) × 4 (letter – national template control, open-ended, time-limited, social norm) × 2 (reminder SMS – yes or no) factorial design was used. The open-ended letter used simplification, behavioural instruction and a personalised planning prompt for patients to record the date and time of their NHS HC. The time-limited letter was similar but stated the NHS HC was due in a named forthcoming month. The social norms letter was similar to the open-ended letter but included a descriptive social norms message and testimonials from local residents and no planning prompt. The outcome measure was attendance at an NHS HC.

**Results:**

Data for 12, 244 invites were analysed. Uptake increased in almost all letter and SMS combinations compared to the control letter without SMS (Uptake 18%), with increases of up to 12 percentage points for the time-limited letter with pre-notification and reminder (Uptake 30%; Adjusted Odds Ratio AOR 1.86; 95% CI 1.45–2.83; *p* < 0.00); 10 percentage points for the open-ended letter with reminder (Uptake 27%; AOR 1.68; 95% CI 1.31–2.17; *p < *0.00) and a 9 percentage point increase using the time-limited letter with reminder (Uptake 27%; AOR 1.61; 95% CI 1.25–2.10; *p <* 0.00). The reminder SMS increased uptake for all intervention letters. The pre-notification did not add to this effect.

**Conclusions:**

This large randomised controlled trial adds support to the evidence that small, low cost behaviourally informed changes to letter-based invitations can increase uptake of NHS HCs. It also provides novel evidence on the effect of SMS reminders and pre-notification on NHS HC attendance.

**Trial registration:**

Retrospectively Registered (24/01/2014) ISRCTN36027094.

**Electronic supplementary material:**

The online version of this article (10.1186/s12889-019-7476-8) contains supplementary material, which is available to authorized users.

## Background

The NHS Health Check (NHS HC) programme is a population level intervention in England, aimed at reducing the incidence of major cardiovascular disease events. Introduced in 2009, all adults aged 40–74 years, who do not have an excluding medical condition, are invited to attend an NHS HC once every five years. The NHS HC can be used to identify individuals at risk of heart disease, stroke, kidney disease, diabetes and certain types of dementia and offer advice and treatment related to the lifestyle factors that contribute to these conditions, such as obesity, smoking and alcohol consumption [1].

With full coverage, it is estimated that 650 deaths and 9500 non-fatal myocardial infarctions and strokes could be prevented each year as a result of the NHS HC [2]. These figures were modelled on an expected uptake rate of 75% of the eligible population [2, 3]. However uptake was only 48.5% from March 2013 to December 2017 [4]. High participation is key to the programme’s success. The low coverage is concerning both for its effectiveness and ultimate sustainability [5]. Improving uptake is therefore a priority for Public Health England (PHE), who aim for an interim uptake of 66%, ultimately heading towards an ideal of 75% [6].

Letters are the usual method of invitation and PHE produces a national template letter that local areas can choose to use. Some local authorities invite patients by telephone and, anecdotally, some are also now using mobile phone short message service (SMS), in combination with a letter. Many areas also provide opportunistic invitations for example offering health checks in supermarkets or workplaces.

There is a paucity of research that explores ways to increase NHS HC uptake, although more research in this area is beginning to emerge. Failure to recall receiving letters is reported as an issue and use of reminders may have a role for improving uptake of the NHS HC [7]. The use of SMS is emerging as an effective approach for sending reminders and is being used more often within screening and other health care programs [8–10]. There is also evidence supporting cost-effectiveness and greater attendance for health care appointments [11]. Generally, SMS is also perceived as a preferred method of invitation [12–14].

Alternatively, there is evidence to support some form of pre-notification making the arrival of the invitation more salient as observed in colorectal cancer screening studies [15–17] where their use appears to be cost-effective [18].

There is wider evidence from the behavioural science literature showing that the content of letters can have an important impact on behavioural outcomes, such as increasing tax compliance [19], reducing household energy consumption [20], reducing GP antibiotic prescribing [21] and increasing enrolment at family weight-management services [22]. These letters used descriptive social norms, based on the idea that individuals are more likely to do something if they believe that others are also doing it. In the NHS HC literature, Sallis et al. amended the content of the standard template letter and achieved an increase in NHS HC uptake compared to the standard letter control [23]. The enhanced letter used simplification (i.e. shorter), behavioural instruction (a clear prompt to call and book an appointment), increased personal salience (‘Your NHS HC is *due’*) and also included a planning prompt in the form of a tear off slip to record the date, time and location of the NHS HC appointment aimed at overcoming the intention-behaviour gap [24]. Planning prompts have also been demonstrated to increase attendance at colonoscopies through use of a patient letter including the opportunity to write the date of their appointment and who it was with [25].

Norman and Conner found that patients were more likely to attend a general health check when their invitation letter contained a pre-booked appointment than when it contained an open-ended invitation [26]. This is also reflected in the screening literature, where the authors of a meta-analyses found that patients were more likely to attend a screening appointment when they are sent a letter that offers a fixed appointment rather than an open-ended invitation, for both cervical screening [27] and across screening programmes in general [28]. Similarly, in the domain of flu vaccinations, clinics that offered only one available day for individuals to receive a flu vaccination had higher vaccination rates than clinics that offered availability on three or five days [29]. This implies that invitations may be most effective when opportunities for attendance are limited. Cialdini suggests that applying a limit to the availability of a product or service gives the impression that it is scarce and therefore increases its perceived value [30].

We sought to provide robust evidence for the impact of different letter content and SMS pre-notification and reminders on participation in the NHS HC.

## Methods

In this research, we aimed to increase uptake of NHS HCs in the inner London Borough of Southwark using invitation letters including (i) open-ended or (ii) time-limited appointment slots, with planning prompts, simplification, behavioural instruction and personal salience or (iii) social norms with simplification, behavioural instruction and personal salience, all sent alongside SMS pre-notification and reminders.

### Study design

**Fig. 1:**
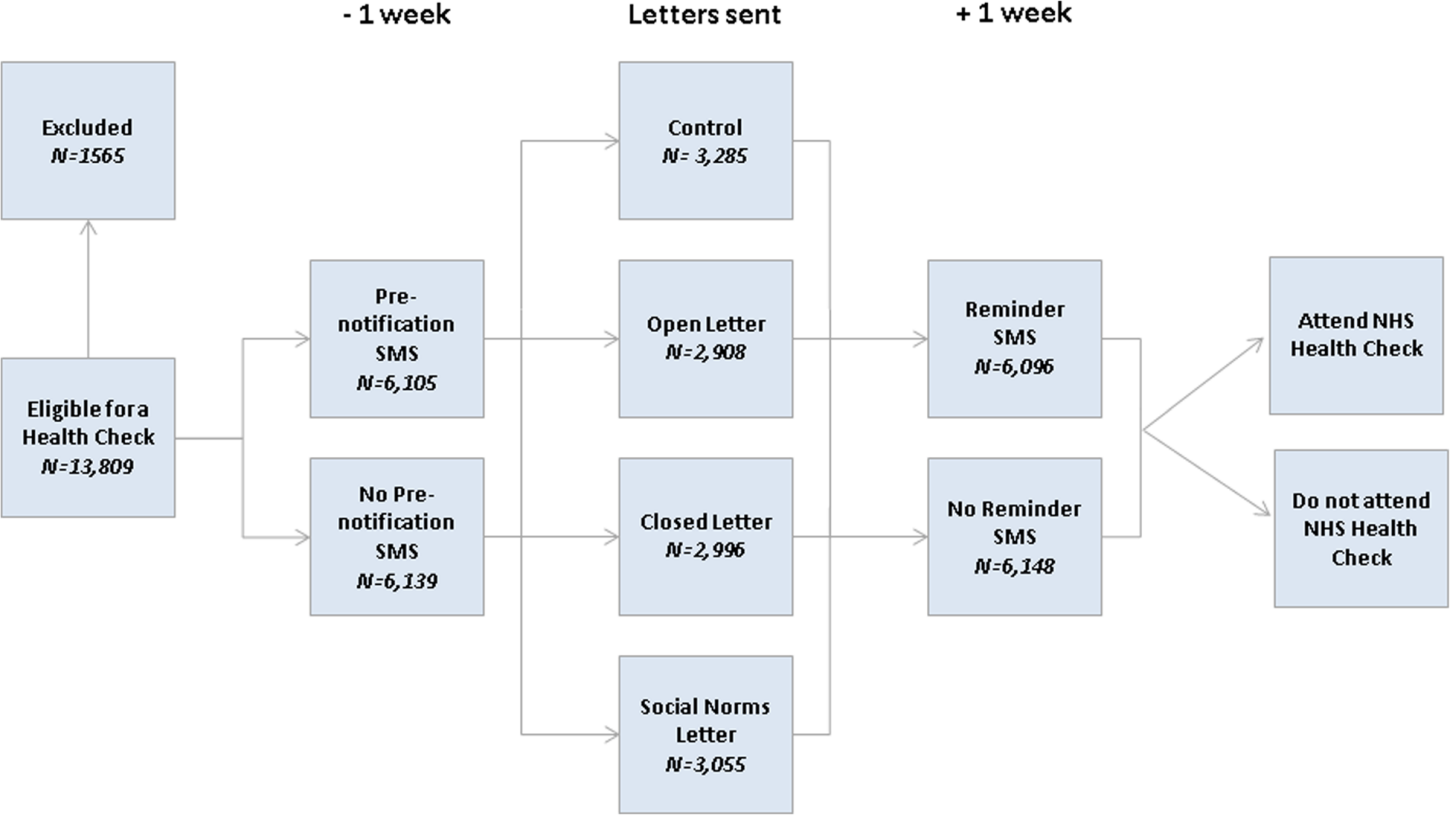
Factorial Design

This study was a double-blind randomised controlled trial with a mixed 2 (pre-notification SMS: yes or no) × 4 (letter: control, open-ended, time-limited, social norm) × 2 (reminder SMS: yes or no) factorial design (Fig. 1). The study was approved by the NHS National Research Ethics Service after proportionate review on 24th October 2013 - REC 13/SW/0293.

### Participants

All 45 General Practices in the London Borough of Southwark were invited to take part via an email from the public health department at Southwark Council. Four were subsequently excluded as they only offered the NHS HC through pharmacies. Twenty-eight practices agreed to take part and submitted an online consent form. Participants were individuals with records on the IT systems at any of these practices who were eligible to receive an NHS HC between 1st November 2013 and 31st December 2014. To be eligible to receive an NHS HC, an individual must have no pre-existing vascular condition and be aged between 40 and 74 years. Patients in Southwark are invited once every five years in the month of their quinquennial birthday (40, 45, 50 etc.). This continued to be the case during the trial. Participants were not required to give consent.

### Randomisation and masking

Data on eligible patients from the 28 practices were extracted from the centralised Southwark Council database, managed by Quality Management Systems (QMS), and divided by general practice on a monthly basis. Primary Care Support Service (PCSS) extracted the patient data and sent it to individual practices to approve. Patient records were then allocated to combinations of SMS interventions (pre-notification and reminder, pre-notification only, reminder only or no SMS) using simple randomisation, whereby each patient had an equal probability of being in any of the four conditions. This was done by iPlato using Mersenne Twister, which is a pseudo-random number generator, an algorithm for generating a sequence of numbers whose properties approximate the properties of sequences of random numbers [31]. Patients without mobile phone numbers recorded in the database were included in the randomisation but subsequently excluded from analysis. QMS then allocated each patient to a letter (control, open-ended, time-limited, social norm) based on the number that was generated. The employees at iPlato and QMS who conducted the randomisation were aware of the interventions being performed in each condition. Staff in the general practices were unaware of allocation and patients were not aware of conditions other than their own. Demographic data were collected by the NHS HC programme manager, according to established NHS information governance standards.

### Procedure

Each patient was invited to have an NHS HC once during the study period. Batches of invitation letters and SMS were sent out on a monthly basis to patients directly from a centralised system. Patients allocated to the pre-notification group were sent an SMS one week before their appointment letters. Reminder SMS were sent one week after the letter. A standard reminder letter was sent 12 weeks after the invitation letter. PCSS managed the letter process, while iPlato managed the SMS process. Both transferred the data after randomisation to the researchers, who merged it using a unique identifier.

### Interventions

There were four letters. The control letter was the existing letter used in Southwark (see Additional file 1), based on the national template letter. The first intervention letter, the *open-ended letter* (see Additional file 2), was an open-ended invitation to book a health check, which was simplified by reducing content from seven to two or three short paragraphs. Simplification of this nature is used as humans can only perceive and attend to a limited amount of information from our environments to make decisions. We employ mental short-cuts to reduce the cognitive effort required to process information – we are cognitive misers [32]. The letter included behavioural instruction asking patients to ‘call to book an appointment’, designed to instruct the individual as to what action they needed to take in simple and concrete language. It also included an opening statement that read ‘Your NHS Health Check is now due’. The use of the word ‘due’ aims to increase the intensity and personal relevance of the perceived expectation to book, as well as implicitly suggesting that the default action is to book an appointment. The open-ended letter also included a personalised planning prompt in the form of a tear-off slip for patients to record the date and time of their NHS HC and then place it somewhere it would remind them to attend. These changes replicate Sallis et al., [23] apart from the additional element of personalisation added to the planning prompt—the person’s name, GP practice and practice address were all pre-filled with a mail merge.

The second intervention letter, the *time-limited letter* (see Additional file 3), was the same as the open-ended letter, but offered a time limited appointment slot – ‘Your NHS Health Check is due in March’.

The final intervention letter, the *social norms letter* (see Additional file 4), was similar to the open-ended letter, but also included a descriptive social norms message about thousands of people attending their health check in Southwark and testimonials from local residents. Importantly, similarity was emphasised—‘thousands of people like you have attended their health check’. This is because the greater the perceived similarity between the described norm and the target, the more effective it has been shown to be [33]. Testimonials were added to this letter as they are commonly used in marketing and we wanted to understand if this approach is effective in this context potentially enhancing the impact of the social norms message. This letter did not have a planning prompt. The four letters can be found in the Supplementary Materials. The behavioural insights and behavior change techniques [34] used in each letter are summarised in Table 1.

**Table 1:** Behavioural insights used in the three intervention letters

Behavioural insight	Intervention Letter		
	Open-ended letter	Time-limited letter	Norms letter
Simplification (shorter letter compared to control)	X	X	X
Behavioural instruction (BCT: 4.1 Instruction on how to perform the behaviour)	X	X	X
Personalised planning prompt i.e. tear-off slip (BCT: 1.4 Action Planning & 7.1 Prompt/cue)	X	X	
Personal salience (Your NHS HC is due)	X		X
Time limited appointment slot (i.e. Your NHS HC is due in March)		X	
Social norms message and testimonials (BCT: 6.2 Social comparison)			X

SMS pre-notification and reminder messages were also used. This study tested the presence or absence of a pre-notification or reminder SMS and not the content of these messages. The SMS content was as follows:

#### Pre-notification

<Practicename>: Dear <firstname2>, your NHS Health Check is due at your GP practice. We will post you a letter soon with info about how to book your appt.

#### Reminder text

<Practicename>: Dear <firstname2>, Your GP recently sent you a letter inviting you to attend your NHS Health Check. Call xxxxxxxxx to book an appt.

### Outcomes

The primary outcome measure was attendance at an NHS HC, recorded as a completed NHS HC by a Read code in patients’ electronic records, and extracted at the end of the study. Secondary outcomes were the effect of age, sex, deprivation quintile, ethnicity (white, black, Asian, other, and not coded/ not known), and GP practice on uptake.

### Statistical analysis

A power calculation was conducted a priori to estimate the required sample size. To detect an effect size of 4 percentage points, with a power of 80% and alpha level of 5%, the required sample size was estimated to be 12,563, or 3141 per letter condition. Statistical analysis was performed in Stata SE 12.0. Data were excluded where mobile phone numbers were not available. Chi-squared tests for categorical variables (sex, ethnicity and deprivation quintile) and one-way analysis of variance for the continuous variable (age) were used to determine if there were any statistically uneven distribution of invitation methods by demographic factors (Table 2). Odds ratios were calculated to compare the likelihood of uptake in each of the 15 conditions against the likelihood of uptake in the control condition— no pre-notification, control letter and no reminder (Table 3). Mixed effects logistic regression models were used to improve inferential power in comparing intervention combinations. The models included patients’ demographical variables as fixed effects and the practice as a random effect in addition to intervention method combinations. The results of the final model are given in Table 4.

**Table 2:** Number and percentage of invitations sent out according to the method of invitation

		No pre-notification	SMS pre-notification	*p*-value^a^	Control letter	Open-ended letter	Time-limited letter	Social norms letter	*p*-value	No reminder	SMS reminder	*p*-value
Sex [n (%)]	Female	2771 (45.1)	2754 (45.1)	0.976	1402 (42.7)	1347 (46.3)	1375 (45.9)	1401 (45.9)	0.012	2785 (45.3)	2740 (45.0)	0.696
	Male	3368 (54.9)	3351 (54.9)		1883 (57.3)	1561 (53.7)	1621 (54.1)	1654 (54.1)		3363 (54.7)	3356 (55.1)	
Age at initiation [mean (s.d.)]	Years	47.9 (8.1)	48.0 (8.1)	0.645^b^	48.1 (8.2)	47.8 (8.0)	47.8 (8.0)	47.9 (8.2)	0.312^b^	48.0 (8.14)	47.8 (8.04)	0.298^3^
Ethnicity [n (%)]	White	2584 (42.1)	2654 (43.5)	0.035	1378 (42.0)	1257 (43.2)	1306 (43.6)	1300 (42.6)	0.353	2591 (42.1)	2650 (43.5)	0.106
	Asian	214 (3.5)	261 (4.3)		118 (3.6)	118 (4.1)	111 (3.7)	128 (4.2)		245 (4.0)	230 (3.8)	
	Black	1732 (28.2)	1664 (27.3)		919 (28.0)	816 (28.1)	812 (27.1)	849 (27.8)		1687 (27.4)	1709 (28.0)	
	Other	591 (9.6)	594 (9.7)		309 (9.4)	285 (9.8)	313 (10.5)	278 (9.1)		595 (9.7)	590 (9.7)	
	Not recorded	1015 (16.5)	932 (15.3)		561 (17.1)	432 (14.9)	454 (15.2)	500 (16.4)		1030 (16.8)	917 (15.0)	
Deprivation Quintile [n (%)]	1 – most deprived	2591 (42.2)	2539 (41.6)	0.183	1418 (43.2)	1197 (41.2)	1243 (41.5)	1272 (41.6)	0.097	2587 (42.1)	2543 (41.7)	0.855
	2	2713 (44.2)	2720 (44.6)		1432 (43.6)	1298 (44.6)	1323 (44.2)	1380 (45.2)		2734 (44.5)	2699 (44.3)	
	3	593 (9.7)	644 (10.6)		330 (10.1)	312 (10.7)	292 (9.8)	303 (9.9)		608 (9.9)	629 (10.3)	
	4	216 (3.5)	182 (3.0)		90 (2.7)	91 (3.1)	128 (4.3)	89 (2.9)		194 (3.2)	204 (3.4)	
	5 – least deprived	26 (0.4)	20 (0.3)		15 (0.5)	10 (0.3)	10 (0.3)	11 (0.4)		25 (0.4)	21 (0.3)	
Total		6139 (100)	6105 (100)		3285 (100)	2908 (100)	2996 (100)	3055 (100)		6148 (100)	6096 (100)	

^a^ Chi^2^ test unless otherwise specified ^b^ One-way analysis of variance

**Table 3:** Odds ratios comparing uptake in intervention and control conditions

Pre-notification	Letter	Reminder	Total patients	Uptake (n)	Uptake proportion	Odds ratio	95% Confidence Interval.	
No	control	no	814	148	0.18	1.00		
No	open-ended	no	724	167	0.23	1.35	1.05	1.73
No	time-limited	no	747	158	0.21	1.21	0.94	1.55
No	social norms	no	800	159	0.20	1.12	0.87	1.43
No	control	yes	885	210	0.24	1.40	1.11	1.77
No	open-ended	yes	692	194	0.28	1.75	1.37	2.24
No	time-limited	yes	754	203	0.27	1.66	1.30	2.11
No	social norms	yes	723	176	0.24	1.45	1.13	1.85
Yes	control	no	783	168	0.21	1.23	0.96	1.57
Yes	open-ended	no	765	204	0.27	1.64	1.29	2.08
Yes	time-limited	no	761	197	0.26	1.57	1.24	2.00
Yes	social norms	no	754	179	0.24	1.40	1.10	1.79
Yes	control	yes	803	192	0.24	1.41	1.11	1.80
Yes	open-ended	yes	727	179	0.25	1.47	1.15	1.88
Yes	time-limited	yes	734	220	0.30	1.93	1.52	2.44
Yes	social norms	yes	778	190	0.24	1.45	1.14	1.85

Reference category: control letter with no pre-notification or reminder

**Table 4:** Mixed effects logistic regressions with practice as a fixed effect showing combinations of invitation methods

Parameter	Estimate (β)	Standard Error	*Z*-value	*P*-value	Odds Ratio (OR)	95% Confidence Interval for OR Lower Upper	
Variation between Practice	0.190	0.04	2.96	0.002			
Intercept	−1.750	0.11	−13.14	.000			
NoPre_Open-ended_Rem	0.521	0.129	4.03	0.000	1.684	1.307	2.170
NoPre_Open-ended_NoRem	0.248	0.132	1.88	0.060	1.281	0.990	1.658
NoPre_Timeltd_Rem	0.476	0.128	3.73	0.000	1.609	1.253	2.066
NoPre_Timeltd_NoRem	0.143	0.132	1.08	0.282	1.153	0.890	1.495
NoPre_Social_Norms_Rem	0.339	0.130	2.6	0.009	1.403	1.087	1.812
NoPre_Social_Norms_NoRem	0.077	0.132	0.58	0.560	1.080	0.834	1.397
NoPre_Ctr_Rem	0.302	0.125	2.42	0.016	1.353	1.059	1.728
Pre_Open-ended_Rem	0.325	0.130	2.5	0.012	1.384	1.073	1.786
Pre_Open-ended_NoRem	0.416	0.127	3.27	0.001	1.516	1.182	1.945
Pre_Timeltd_Rem	0.620	0.127	4.91	0.000	1.860	1.451	2.383
Pre_Timeltd_NoRem	0.377	0.128	2.95	0.003	1.457	1.134	1.872
Pre_Social_Norms_Rem	0.316	0.128	2.47	0.014	1.372	1.067	1.764
Pre_Social_Norms_NoRem	0.306	0.130	2.36	0.018	1.358	1.053	1.752
Pre_Ctr_Rem	0.313	0.128	2.45	0.014	1.367	1.065	1.756
Pre_Ctr_NoRem	0.213	0.131	1.63	0.104	1.237	0.958	1.598
GENDER (Ref: Female)	−0.314	0.044	−7.09	0.000	0.730	0.670	0.797
Asian (Ref: White)	0.006	0.113	0.06	0.955	1.006	0.807	1.255
Black	0.255	0.053	4.79	0.000	1.291	1.163	1.433
Other	−0.087	0.078	−1.11	0.268	0.917	0.786	1.069
Not stated/not known	−2.322	0.120	−19.36	0.000	0.098	0.078	0.124
Dep Quintile 2 (Ref: Quintile 1)	0.015	0.052	0.29	0.772	1.015	0.917	1.123
Dep Quintile 3	0.040	0.092	0.43	0.667	1.041	0.868	1.247
Dep Quintile 4	−0.150	0.166	−0.91	0.365	0.860	0.622	1.191
Dep Quintile 5	−0.192	0.389	−0.49	0.621	0.825	0.385	1.768
Dep Quintile Unknown	0.212	0.419	0.51	0.613	1.236	0.544	2.810
Age at invitation	0.012	0.003	4.49	0.000	1.012	1.007	1.018

Residual maximum likelihood estimate of model parameters (*β*), *Pre* = Pre-notification, *Rem* = Reminder; *Ctr* = control letter, *Open-ended* = Open-ended letter, *Timeltd* = Time-limited letter, *Social_Norms* = Social norms letter

Reference category: no prenotifcaiton, control letter and no reminder

## Results

### Descriptive statistics

Between 4th December 2013 and 24th November 2014, 13,809 invitation letters were sent. Of these invitations, 1565 were excluded from the analysis because there was no valid mobile phone number available. Of the 12,244 invitations included in the analysis, 3285 (26.9%) patients were sent the control letter, 2908 (23.8%) the open-ended letter, 2996 (24.5%) the time-limited letter, and 3055 (25.0%) the social norms letter (Table 2). Across these four groups 3085 (25.20%) received no SMS; 3063 (25.02%) received a pre-notification SMS only, 3054 (24.94%) received a reminder SMS only and 3042 (24.84%) received both a pre-notification and reminder SMS. More than half the invitees were male (54.9%), the largest ethnic group was white (42.8%), although a substantial percentage of patients were black (27.8%) or had no ethnicity stated (15.9%), and 86.1% of the invitees were in the lowest two quintiles of deprivation by area of residence (0.3% could not be assigned a deprivation quintile). There were two significant differences in the allocation of invitees across trial arms according to these demographic factors (see Table 2). There was a larger proportion of men in the control letter trial arm (57.3%) (*p* = 0.012). There was also a slightly different distribution by ethnic group between the trial arms that were sent the SMS pre-notification or not (*p* = 0.035).

### Uptake of health checks

**Fig. 2:**
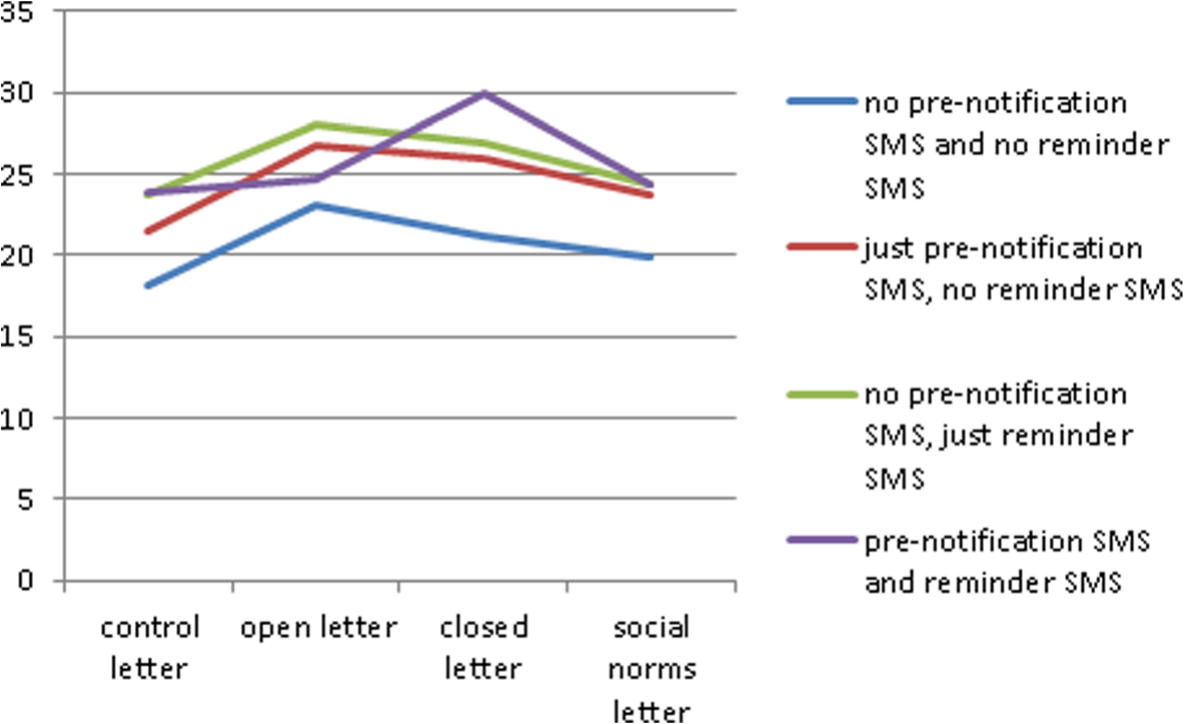
NHS Health Check Uptake

Overall, 24% (2944) of the 12,244 invitees included in the analysis attended an NHS HC between 5th December 2014 and 2nd March 2015 (Table 3). The percentage of those invited who had an NHS HC (uptake) varied from 18.2% for those who received the control letter without pre-notification or reminder SMS to 30.0% for those who received the time-limited letter with pre-notification and reminder SMS (see Fig. 2).

To identify which combinations of interventions increased the likelihood that patients would attend their NHS HCs, odds ratios were calculated, comparing the odds of uptake of an NHS HC in each intervention combination with the odds of uptake in the control condition (no pre-notification, control letter, no reminder). The 95% confidence intervals for odds ratios in the intervention conditions were greater than one in all but three conditions: time-limited letter with no SMS, social norm letter with no SMS, and the control letter with pre-notification but no reminder suggesting all other intervention combinations increased uptake (Table 3).

These results are statistically confirmed by a mixed effects logistic regression model including patients’ demographic variables (sex, ethnicity and deprivation quintile) as fixed effects and the practice as a random effect; using intervention method combinations as the independent variables, and the baseline combination of no pre-notification SMS, control letter, and no reminder SMS (Table 4). The results indicated that apart from four intervention combinations (all intervention letters without an SMS and the control letter with pre-notification), patients from all 11 remaining intervention combinations were more likely to attend an NHS HC than those in the control. The time-limited letter with pre-notification and reminder texts was the most effective intervention combination (Adjusted Odds Ratio (AOR) 1.86; 95% CI 1.4–2.38; *p* < 0.00). Open-ended or time-limited letters with a reminder text had the next largest odds ratios (AOR 1.68; 95% CI 1.31–2.17; p < 0.00 and AOR 1.61; 95% CI 1.25–2.07; p < 0.00). Testing all combinations simultaneously, the model suggests a significant variation between the intervention combinations on attending NHS HC (Wald statistic 47.12; 15 degrees of freedom; p < 0.00).

For the 15 intervention combinations in Table 4, there are 105 possible pairwise comparisons. (Additional file 5). Amongst the pairwise comparisons there were only 21 pairs with associated 95% confidence intervals that did not include 1, indicating that the two combinations being compared led to differential uptake. These comparisons indicate that the reminder SMS was successful in increasing uptake of the NHS HC when it was the only text sent alongside any of the three invitation letters. This was indicated by the significant difference in pairwise comparisons between conditions with or without the reminder for the open-ended letter (OR 0.76; 95% CI 0.59–0.98); time-limited letter (OR 0.72; 95% CI 0.56–0.92); and social norms letter (OR 0.77; 95% CI 0.60–0.99). Although the time-limited letter with pre-notification and reminder texts had the highest odds ratio, there was not enough statistical evidence to suggest differences in NHS HC uptake probability when using a pre-notification SMS, in particular when it was the only text sent. Non-significant pairwise comparisons indicated that the reminder SMS alone was successful in increasing uptake of the NHS HC when it was the only text sent alongside any of the three invitation letters and pre-notification was not necessary. This was indicated by the non-significant difference between letter conditions with or without pre-notifications —open-ended letter (OR 1.22; 95% CI 0.95–1.56); time-limited (OR 0.87; 95% CI 0.68–1.10); and social norm (OR 1.02; 95% CI 0.80–1.31).

**Fig. 3:**
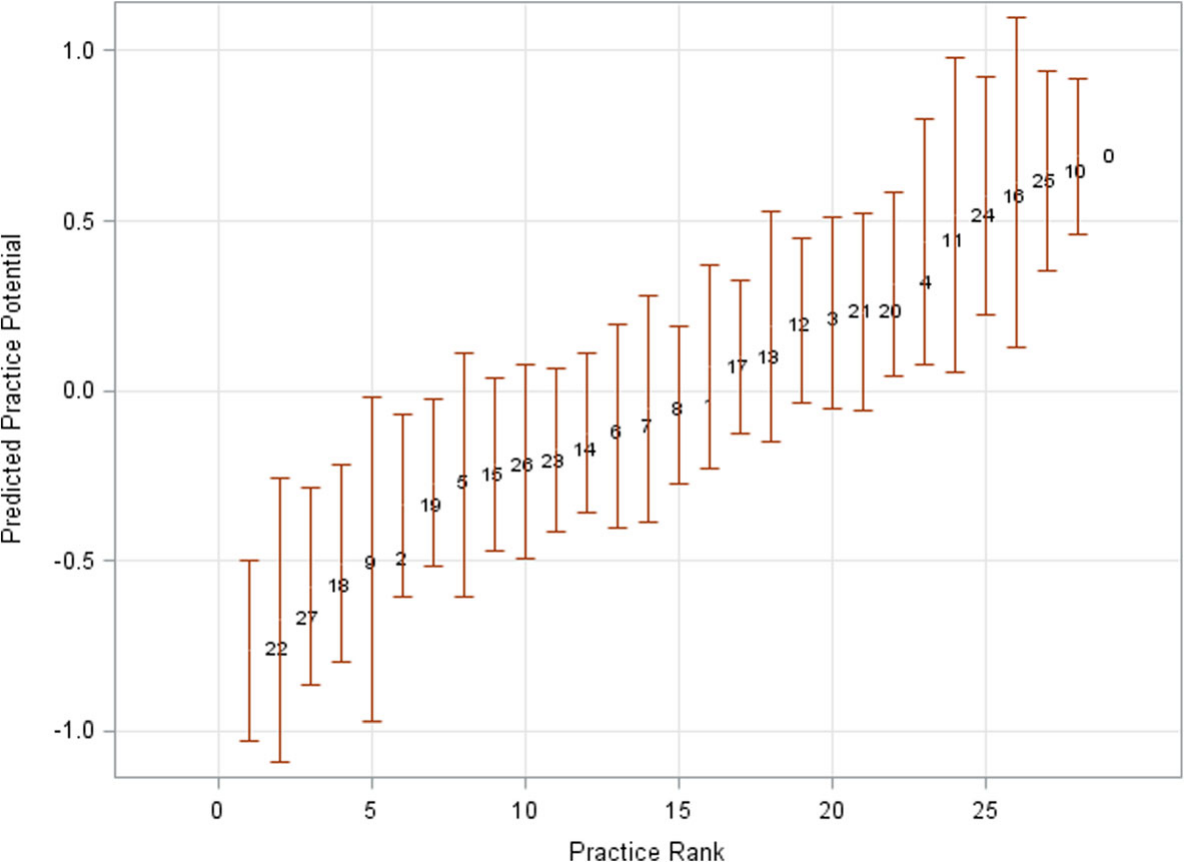
Predicted values of the practice random effects

Demographic variables affected uptake (see Table 4). The likelihood of attending an NHS HC increased with age (OR 1.01; 95% CI 1.01–1.02, *p* < 0.00). Male patients were less likely to attend an NHS HC than females (OR 0.73; 95% CI 0.67–0.80; p < 0.00). There was also a significant difference in the ethnicity of people who had an NHS HC. Those who gave their ethnicity as black were more likely to attend than those who reported their ethnicity as white. Furthermore those whose ethnicity was not stated or not known were less likely to attend than those whose ethnicity was white. Deprivation was also included in the final estimated model and there was no support for an effect based on deprivation (Wald statistic 1.98; 5 degrees of freedom, *p* = 0.85). We might expect that deprivation is correlated with practice so any effect of deprivation would be absorbed into the practice effect. There was a significant variation in uptake between practices. Figure 3 shows the predicted values of unknown practice random effects. The predicted values of practice random effects indicated differences between practices in relative uptake of the NHS HC. If there was no variation then they would all be at zero. However, they are spread between − 1 and 1; seven practices had patients who were more likely to attend a health check and seven had patients who were less likely to have an NHS HC.

## Discussion

In all but four intervention combinations, the likelihood of attending an NHS HC was higher compared to the control letter without SMS pre-notification or reminder. There were large increases in uptake of up to 12 percentage points for the time-limited letter with pre-notification and reminder compared to control. The next most effective combination was the open-ended letter with a reminder and no pre-notification (10 percentage points) followed by the time-limited letter with a reminder and no pre-notification (9 percentage points). Reminder texts were effective at increasing uptake of the NHS HC alongside all intervention letters. There was not enough statistical evidence to suggest the use of pre-notification SMS in addition to reminders.

The time-limited letter was successful in increasing uptake of the NHS HC in various combinations, consistent with other evidence that providing a limited time period in which to attend an appointment will increase uptake [26–29, 35] . The time-limited letter was very similar to the open-ended letter (both were shorter than the standard letter and included a personalised planning prompt and clear behavioral instruction to call and book an appointment) with the addition that it stated that the NHS HC was due in the forthcoming named month. It is possible that the open-ended and time-limited letters did not differ substantially in their effects on uptake because the time limited appointment slot was too wide, allowing a whole month for attendance. This may have potentially reduced the urgency compared to the open-ended letter which suggested the patients’ NHS HC is due **‘**now’. Other interventions using this method offered shorter deadlines (i.e. 1 day) [35] which it was not possible to do in this study for practical reasons concerned with NHS HC delivery.

The effectiveness of the open-ended letter is supported by its successful use in Medway, where it increased absolute uptake by 4.2% compared to the national template control letter [23]. This is similar to the 5% increase in uptake in this trial in the combination where patients received the open-ended letter without a pre-notification or a reminder SMS. The open-ended letter used in Medway only differed in that the included planning prompt was not pre-populated with the person’s name and GP address like in the present study. The different contexts preclude our ability to suggest the additional effects are due to the increased personalisation.

Together, the success of the open-ended letter with planning prompt in Medway [23] and the open-ended and time-limited letters with personalised planning prompts in this trial provide good evidence for the use of planning prompts in NHS HC invitation letters. The present research also supports findings from other studies using planning prompts to increase attendance at flu vaccination clinics by recording the date and time of the appointment (4.2 percentage points compared to control) [35] and increasing voter turnout via a phone prompt to write down the time they will vote, where they will be coming from and what they would be doing prior to voting (4.1 percentage points compared to control) [36]. Effect sizes for these studies and the present study are remarkably similar. To increase these effects, further planning prompts could attempt to prompt specific plans to overcome known barriers to attendance such as time off work to attend appointments [37] or increase planning specificity further with the aim of more in-depth reflective processing leading to a stronger link between plans and future actions, thereby reducing likelihood of procrastination and forgetfulness at the time of the event.

Although the social norms letter was more effective than control when combined with an SMS it did not perform as well as open-ended and time-limited letters which is surprising given the success of social norm interventions in other areas (see [19, 21]). It is possible that the message ‘thousands of people like you have attended their health check in Southwark’ is not personalised enough. There is evidence to suggest that the more personalised the norm, the more effective it is [33]. It is also possible that the testimonials looked like marketing materials, making the letter look more like junk mail. It is of note that this less successful letter did not include a planning prompt.

The positive effect of SMS reminders on uptake of the NHS HC is consistent with evidence that they increase attendance at screening programmes. There was less evidence for the effect of SMS pre-notifications on NHS HC uptake in contrast to evidence on colorectal cancer screening [15–17]. The superior effect for reminders may be because they are more effective at bridging the intention-behaviour gap, since they are received after the invitation letter and therefore presumably after the intention to make and attend an appointment has been formed. Reminders also suggest proactive behaviour to book an appointment whereas there are no direct actions associated with pre-notifications. The authors of another recent factorial study explored combinations of behaviourally informed pre-notification and reminder texts to increase return of self-sampling HIV kits [38]. The most effective combination was the behaviourally informed primers and reminders compared to standard reminders – a 4 percentage point increase was observed. But, like the present study, despite a primer (or pre-notification) being included in the most effective combination, further analysis demonstrated that only the behaviourally informed reminders were independently effective and not the primers [38].

Although not the primary aim of the study, it was possible to observe variation in attendance according to sex, age and ethnicity. As with other studies of NHS HC uptake, the likelihood of attendance increased in female patients [23, 39–42] and with increasing age [23, 39, 40, 42]. Our findings that those with black ethnicity were more likely to attend and that those whose ethnicity is not recorded were less likely to attend are in accordance with the results of other studies [5, 43]. However, findings on the relationship between ethnicity and attendance at health checks are not consistent, and the authors of other studies have found that those with white ethnicity are no more likely to attend than other ethnicities [41]. In the present study we found no relationship between being South East Asian and attending an NHS HC, which differs from other studies [5, 42, 43]. There appears to be significant practice effects and any effect from deprivation score disappears when practice is included as a random effect, suggesting that any deprivation effect is absorbed by the practice effect. A similar practice level disparity has been found in previous trials on uptake of NHS HC [23, 40, 42], some of which found that uptake varies by practice size, being lower for smaller practices [42].

This study has some limitations. It was carried out with only one borough within central London. As such, the sample and setting are not nationally representative, although there is no reason to believe that people in different areas would respond differently to these interventions. It appears that variation between practices is a stronger influence on uptake. Around 1500 patients who did not have mobile numbers were excluded from the trial. It is possible that this group responded differently to the invitation letters from the patients who were included in the trial, or that they came from a particular demographic (e.g. older, more deprived). However, the number of exclusions was relatively small compared to the total number of participants in the trial and they were randomly distributed across the intervention combinations, so they are not expected to have a large effect on the results. There were also differences in demographics across the intervention groups, but when demographics were introduced into the model they did not make much difference to the main effects. The reason for practice variation is also not explored.

The uptake rate even in the best condition of this study remained lower than average (48.5%) suggesting other techniques should be used alongside letters and SMS. One study using observational cohort methodology showed that if a patient is invited by phone or approached verbally in-practice, compared to a letter alone or in addition to a letter, they are three times more likely to attend an NHS HC [39]. Similarly, amongst 30 GP practices in Luton, England highest uptake was found for verbal face-to-face NHS HC offers (71.9%), followed by telephone invitation (43%) and finally by letter invitation (29.5%) [44]. More recently Gidlow et al. compared uptake using the current national template letter (i.e. the open-ended letter used in this study which has since been adopted as the national template) (30.9%) to personalised risk letters (31.3%) and telephone invitations (47.6%) and found only the latter to increase uptake, until multi-level modelling was used, taking account of practice effects which suggested the personalised risk letter to be four percentage points higher than the national template [45]. This suggests that practices could consider optimising the national template letter with personalised risk information if this is available locally.

## Conclusion

This large randomised controlled trial adds further support to the evidence that small, low cost behaviourally informed changes to letter-based invitations can have a substantial impact on NHS HC uptake. There was also an additive effect of an SMS reminder and pre-notification did not add to this effect. The current national template letter recommends use of the open-ended letter in this study and directs local authority staff to the findings of this study to enable local areas to consider additionally the use of pre-notifications and reminders depending upon local resources.

## Additional files

Additional file 1: Control letter. (DOCX 15 kb)

Additional file 2: Open ended letter. (DOC 68 kb)

Additional file 3: Time limited letter. (DOC 68 kb)

Additional file 4: Social norms letter. (DOC 59 kb)

Additional file 5: Pairwise comparisons. (DOCX 16 kb)

## Data Availability

Data for this study is based on patient-level information collected by local authorities, as part of the NHS HC programme. Requests for the data need to be made via PHE’s office for data release.

## Abbreviations

AOR: Adjusted Odds Ratio
GP: General Practitioner
NHS HC: National Health Service Health Check
PCSS: Primary Care Support Service
PHE: Public Health England
QMS: Quality Management Systems
SMS: Short message service
