# Supplementary material for: Pre-notification and reminder SMS text messages with behaviourally informed invitation letters to improve uptake of NHS Health Checks: a factorial randomised controlled trial
Source: BMC Public Health. 2019 Aug 22;19:1162. doi: 10.1186/s12889-019-7476-8 (PMC6706889; doi:10.1186/s12889-019-7476-8)
Supplement: Supplementary file 1 — Control letter. (DOCX 15 kb) [file 12889_2019_7476_MOESM1_ESM.docx]

Ref: 123456

NHS NO: 1234567890

20 September 2013

**Ref: CHD**

Mr Test Patient On behalf of

1^st^ Line Sample Surgery

2^nd^ Line

3^rd^ Line

4^th^ Line

POSTCODE

Dear Mr Test Patient

**Invitation to a free health check**

We are inviting you to have a free NHS Health Check. NHS Health Check is a national programme for people between 40 and 74.

The aim of the check is to assess your risk of developing heart disease, stroke, kidney disease or diabetes and then to work with you to reduce that risk. If we find any problems with your health we can also prescribe treatment.

The check should take about 20-30 minutes and is based on straightforward questions and measurements such as age, sex, family history, height, weight and blood pressure. We will also take a simple blood test to measure your cholesterol and glucose levels, in some cases.

Following the check, you will receive free personalised advice about what you can do to stay healthy, as well as treatment if this is necessary. There is good evidence that by taking early action, you can improve your health and reduce your likelihood of developing these conditions.

**You can have your health check at a local pharmacy listed in the enclosed leaflet or at your surgery (Sample Surgery). To book your check please find 0203 4039 9999 and quote “NHS Health Check”.**

If you are unable to make it to the surgery you can also have your check done by the Southward’s Health Check Outreach Team. Please go to [www.southwarkpct.nhs.uk](http://www.southwarkpct.nhs.uk) and click on Health Living for information on outreach session in Southwark.

Take a look at the enclosed leaflet for more information about the NHS Health Check and how it could benefit you.

Yours sincerely

Dr Doctor
