# Supplementary material for: Pre-notification and reminder SMS text messages with behaviourally informed invitation letters to improve uptake of NHS Health Checks: a factorial randomised controlled trial
Source: BMC Public Health. 2019 Aug 22;19:1162. doi: 10.1186/s12889-019-7476-8 (PMC6706889; doi:10.1186/s12889-019-7476-8)
Supplement: Supplementary file 2 — Open ended letter. (DOC 68 kb) [file 12889_2019_7476_MOESM2_ESM.doc]

Dear <to be inserted by mail merge>

**Your NHS Health Check is now due.**

Please call<to be inserted by mail merge> to book your appointment at your GP’s surgery and record this on the tear off slip below.

You can also have your health check at your local pharmacy listed in the enclosed leaflet. To book, please ring 0203 4039 9999 and quote ‘NHS Health Check’.

Yours sincerely

Dr <to be inserted by mail merge>
