# Supplementary material for: Pre-notification and reminder SMS text messages with behaviourally informed invitation letters to improve uptake of NHS Health Checks: a factorial randomised controlled trial
Source: BMC Public Health. 2019 Aug 22;19:1162. doi: 10.1186/s12889-019-7476-8 (PMC6706889; doi:10.1186/s12889-019-7476-8)
Supplement: Supplementary file 3 — Time limited letter. (DOC 68 kb) [file 12889_2019_7476_MOESM3_ESM.doc]

Dear <to be inserted by mail merge>

**Your NHS Health Check is due in August.**

Please call <to be inserted by mail merge> as soon as possible to make sure you get your appointment at your GP’s surgery and record this on the tear off slip below.

You can also have your health check at your local pharmacy listed in the enclosed leaflet. To book, please ring 0203 4039 9999 and quote ‘NHS Health Check’.

Yours sincerely

Dr <to be inserted by mail merge>
