# Supplementary material for: Pre-notification and reminder SMS text messages with behaviourally informed invitation letters to improve uptake of NHS Health Checks: a factorial randomised controlled trial
Source: BMC Public Health. 2019 Aug 22;19:1162. doi: 10.1186/s12889-019-7476-8 (PMC6706889; doi:10.1186/s12889-019-7476-8)
Supplement: Supplementary file 4 — Social norms letter. (DOC 59 kb) [file 12889_2019_7476_MOESM4_ESM.doc]

**How an NHS Health Check helped me**

*“My cholesterol was a little high, but I got peace of mind regarding my state of health generally. Since the health check, I’ve cut down on alcohol and made some small changes to my diet”*

**Mr RK, Southwark**

*“I already knew I was overweight, but having a health check was a wake-up call which helped me understand the risks of developing diabetes and heart disease. I’ve changed my eating habits, eating smaller portions, less sugar and oily food, and more salad and vegetables. I exercise a lot more - I’ve joined an aerobics class and really enjoy going for brisk walks. I’ve already lost nearly half a stone and would definitely advise others to go for it.”*

**Ms CD, Southwark**

Dear <to be inserted by mail merge>

**Your NHS Health Check is now due.**

In Southwark, thousands of people like you have attended their health check and benefited from personalised health advice.

Please call <to be inserted by mail merge>to book your appointment at your GP’s surgery.

You can also have your health check at your local pharmacy listed in the enclosed leaflet. To book, please ring 0203 4039 9999 and quote ‘NHS Health Check’.

Yours sincerely

Dr <to be inserted by mail merge>
