# Supplementary material for: Pre-notification and reminder SMS text messages with behaviourally informed invitation letters to improve uptake of NHS Health Checks: a factorial randomised controlled trial
Source: BMC Public Health. 2019 Aug 22;19:1162. doi: 10.1186/s12889-019-7476-8 (PMC6706889; doi:10.1186/s12889-019-7476-8)
Supplement: Supplementary file 5 — Pairwise comparisons. (DOCX 16 kb) [file 12889_2019_7476_MOESM5_ESM.docx]

Additional file 5: Pair comparison of the invitation methods combinations

| Invitation method combination VS Invitation method combination | | | Odds-ratio | 95 Conf. | |
| --- | --- | --- | --- | --- | --- |
| PRIMER0*LETTER0*REMIND1 | VS. | PRIMER1*LETTER2*REMIND1 | 0.73 | 0.58 | 0.92 |
| PRIMER0*LETTER1*REMIND0 | VS. | PRIMER0*LETTER1*REMIND1 | 0.76 | 0.59 | 0.98 |
| PRIMER0*LETTER1*REMIND0 | VS. | PRIMER1*LETTER2*REMIND1 | 0.69 | 0.54 | 0.88 |
| PRIMER0*LETTER1*REMIND1 | VS. | PRIMER0*LETTER2*REMIND0 | 1.46 | 1.14 | 1.88 |
| PRIMER0*LETTER1*REMIND1 | VS. | PRIMER0*LETTER3*REMIND0 | 1.56 | 1.21 | 2 |
| PRIMER0*LETTER1*REMIND1 | VS. | PRIMER1*LETTER0*REMIND0 | 1.36 | 1.06 | 1.74 |
| PRIMER0*LETTER2*REMIND0 | VS. | PRIMER0*LETTER2*REMIND1 | 0.72 | 0.56 | 0.92 |
| PRIMER0*LETTER2*REMIND0 | VS. | PRIMER1*LETTER1*REMIND0 | 0.76 | 0.59 | 0.97 |
| PRIMER0*LETTER2*REMIND0 | VS. | PRIMER1*LETTER2*REMIND1 | 0.62 | 0.48 | 0.79 |
| PRIMER0*LETTER2*REMIND1 | VS. | PRIMER0*LETTER3*REMIND0 | 1.49 | 1.16 | 1.91 |
| PRIMER0*LETTER2*REMIND1 | VS. | PRIMER1*LETTER0*REMIND0 | 1.3 | 1.02 | 1.66 |
| PRIMER0*LETTER3*REMIND0 | VS. | PRIMER0*LETTER3*REMIND1 | 0.77 | 0.6 | 0.99 |
| PRIMER0*LETTER3*REMIND0 | VS. | PRIMER1*LETTER1*REMIND0 | 0.71 | 0.56 | 0.91 |
| PRIMER0*LETTER3*REMIND0 | VS. | PRIMER1*LETTER1*REMIND1 | 0.78 | 0.61 | 1 |
| PRIMER0*LETTER3*REMIND0 | VS. | PRIMER1*LETTER2*REMIND0 | 0.74 | 0.58 | 0.95 |
| PRIMER0*LETTER3*REMIND0 | VS. | PRIMER1*LETTER2*REMIND1 | 0.58 | 0.45 | 0.74 |
| PRIMER0*LETTER3*REMIND1 | VS. | PRIMER1*LETTER2*REMIND1 | 0.75 | 0.59 | 0.96 |
| PRIMER1*LETTER0*REMIND0 | VS. | PRIMER1*LETTER2*REMIND1 | 0.67 | 0.52 | 0.85 |
| PRIMER1*LETTER0*REMIND1 | VS. | PRIMER1*LETTER2*REMIND1 | 0.74 | 0.58 | 0.93 |
| PRIMER1*LETTER1*REMIND1 | VS. | PRIMER1*LETTER2*REMIND1 | 0.74 | 0.58 | 0.95 |
| PRIMER1*LETTER2*REMIND0 | VS. | PRIMER1*LETTER2*REMIND1 | 0.78 | 0.62 | 0.99 |
| PRIMER1*LETTER2*REMIND1 | VS. | PRIMER1*LETTER3*REMIND0 | 1.37 | 1.08 | 1.74 |
| PRIMER1*LETTER2*REMIND1 | VS. | PRIMER1*LETTER3*REMIND1 | 1.36 | 1.07 | 1.72 |
| PRIMER0*LETTER0*REMIND1 | VS. | PRIMER0*LETTER3*REMIND0 | 1.25 | 0.98 | 1.6 |
| PRIMER0*LETTER1*REMIND0 | VS. | PRIMER0*LETTER3*REMIND0 | 1.19 | 0.92 | 1.53 |
| PRIMER0*LETTER1*REMIND1 | VS. | PRIMER0*LETTER3*REMIND1 | 1.2 | 0.94 | 1.54 |
| PRIMER0*LETTER1*REMIND1 | VS. | PRIMER1*LETTER0*REMIND1 | 1.23 | 0.97 | 1.57 |
| PRIMER0*LETTER1*REMIND1 | VS. | PRIMER1*LETTER1*REMIND1 | 1.22 | 0.95 | 1.56 |
| PRIMER0*LETTER1*REMIND1 | VS. | PRIMER1*LETTER2*REMIND0 | 1.16 | 0.91 | 1.47 |
| PRIMER0*LETTER1*REMIND1 | VS. | PRIMER1*LETTER3*REMIND0 | 1.24 | 0.97 | 1.59 |
| PRIMER0*LETTER1*REMIND1 | VS. | PRIMER1*LETTER3*REMIND1 | 1.23 | 0.96 | 1.57 |
| PRIMER0*LETTER2*REMIND1 | VS. | PRIMER0*LETTER3*REMIND1 | 1.15 | 0.9 | 1.46 |
| PRIMER0*LETTER2*REMIND1 | VS. | PRIMER1*LETTER0*REMIND1 | 1.18 | 0.93 | 1.49 |
| PRIMER0*LETTER2*REMIND1 | VS. | PRIMER1*LETTER1*REMIND1 | 1.16 | 0.91 | 1.48 |
| PRIMER0*LETTER2*REMIND1 | VS. | PRIMER1*LETTER3*REMIND0 | 1.18 | 0.93 | 1.51 |
| PRIMER0*LETTER2*REMIND1 | VS. | PRIMER1*LETTER3*REMIND1 | 1.17 | 0.92 | 1.49 |
| PRIMER0*LETTER0*REMIND1 | VS. | PRIMER0*LETTER1*REMIND0 | 1.06 | 0.83 | 1.34 |
| PRIMER0*LETTER0*REMIND1 | VS. | PRIMER0*LETTER1*REMIND1 | 0.8 | 0.63 | 1.02 |
| PRIMER0*LETTER0*REMIND1 | VS. | PRIMER0*LETTER2*REMIND0 | 1.17 | 0.92 | 1.5 |
| PRIMER0*LETTER0*REMIND1 | VS. | PRIMER0*LETTER2*REMIND1 | 0.84 | 0.67 | 1.06 |
| PRIMER0*LETTER0*REMIND1 | VS. | PRIMER0*LETTER3*REMIND1 | 0.96 | 0.76 | 1.22 |
| PRIMER0*LETTER0*REMIND1 | VS. | PRIMER1*LETTER0*REMIND0 | 1.09 | 0.86 | 1.39 |
| PRIMER0*LETTER0*REMIND1 | VS. | PRIMER1*LETTER0*REMIND1 | 0.99 | 0.78 | 1.25 |
| PRIMER0*LETTER0*REMIND1 | VS. | PRIMER1*LETTER1*REMIND0 | 0.89 | 0.71 | 1.13 |
| PRIMER0*LETTER0*REMIND1 | VS. | PRIMER1*LETTER1*REMIND1 | 0.98 | 0.77 | 1.24 |
| PRIMER0*LETTER0*REMIND1 | VS. | PRIMER1*LETTER2*REMIND0 | 0.93 | 0.74 | 1.17 |
| PRIMER0*LETTER0*REMIND1 | VS. | PRIMER1*LETTER3*REMIND0 | 1 | 0.79 | 1.26 |
| PRIMER0*LETTER0*REMIND1 | VS. | PRIMER1*LETTER3*REMIND1 | 0.99 | 0.78 | 1.25 |
| PRIMER0*LETTER1*REMIND0 | VS. | PRIMER0*LETTER2*REMIND0 | 1.11 | 0.86 | 1.44 |
| PRIMER0*LETTER1*REMIND0 | VS. | PRIMER0*LETTER2*REMIND1 | 0.8 | 0.62 | 1.02 |
| PRIMER0*LETTER1*REMIND0 | VS. | PRIMER0*LETTER3*REMIND1 | 0.91 | 0.71 | 1.17 |
| PRIMER0*LETTER1*REMIND0 | VS. | PRIMER1*LETTER0*REMIND0 | 1.04 | 0.8 | 1.33 |
| PRIMER0*LETTER1*REMIND0 | VS. | PRIMER1*LETTER0*REMIND1 | 0.94 | 0.73 | 1.2 |
| PRIMER0*LETTER1*REMIND0 | VS. | PRIMER1*LETTER1*REMIND0 | 0.85 | 0.66 | 1.08 |
| PRIMER0*LETTER1*REMIND0 | VS. | PRIMER1*LETTER1*REMIND1 | 0.93 | 0.72 | 1.19 |
| PRIMER0*LETTER1*REMIND0 | VS. | PRIMER1*LETTER2*REMIND0 | 0.88 | 0.69 | 1.13 |
| PRIMER0*LETTER1*REMIND0 | VS. | PRIMER1*LETTER3*REMIND0 | 0.94 | 0.73 | 1.21 |
| PRIMER0*LETTER1*REMIND0 | VS. | PRIMER1*LETTER3*REMIND1 | 0.93 | 0.73 | 1.2 |
| PRIMER0*LETTER1*REMIND1 | VS. | PRIMER0*LETTER2*REMIND1 | 1.05 | 0.82 | 1.33 |
| PRIMER0*LETTER1*REMIND1 | VS. | PRIMER1*LETTER1*REMIND0 | 1.11 | 0.87 | 1.41 |
| PRIMER0*LETTER1*REMIND1 | VS. | PRIMER1*LETTER2*REMIND1 | 0.91 | 0.71 | 1.15 |
| PRIMER0*LETTER2*REMIND0 | VS. | PRIMER0*LETTER3*REMIND0 | 1.07 | 0.83 | 1.38 |
| PRIMER0*LETTER2*REMIND0 | VS. | PRIMER0*LETTER3*REMIND1 | 0.82 | 0.64 | 1.06 |
| PRIMER0*LETTER2*REMIND0 | VS. | PRIMER1*LETTER0*REMIND0 | 0.93 | 0.72 | 1.2 |
| PRIMER0*LETTER2*REMIND0 | VS. | PRIMER1*LETTER0*REMIND1 | 0.84 | 0.66 | 1.08 |
| PRIMER0*LETTER2*REMIND0 | VS. | PRIMER1*LETTER1*REMIND1 | 0.83 | 0.65 | 1.07 |
| PRIMER0*LETTER2*REMIND0 | VS. | PRIMER1*LETTER2*REMIND0 | 0.79 | 0.62 | 1.01 |
| PRIMER0*LETTER2*REMIND0 | VS. | PRIMER1*LETTER3*REMIND0 | 0.85 | 0.66 | 1.09 |
| PRIMER0*LETTER2*REMIND0 | VS. | PRIMER1*LETTER3*REMIND1 | 0.84 | 0.66 | 1.08 |
| PRIMER0*LETTER2*REMIND1 | VS. | PRIMER1*LETTER1*REMIND0 | 1.06 | 0.84 | 1.35 |
| PRIMER0*LETTER2*REMIND1 | VS. | PRIMER1*LETTER2*REMIND0 | 1.1 | 0.87 | 1.4 |
| PRIMER0*LETTER2*REMIND1 | VS. | PRIMER1*LETTER2*REMIND1 | 0.87 | 0.68 | 1.1 |
| PRIMER0*LETTER3*REMIND0 | VS. | PRIMER1*LETTER0*REMIND0 | 0.87 | 0.68 | 1.12 |
| PRIMER0*LETTER3*REMIND0 | VS. | PRIMER1*LETTER0*REMIND1 | 0.79 | 0.62 | 1.01 |
| PRIMER0*LETTER3*REMIND0 | VS. | PRIMER1*LETTER3*REMIND0 | 0.79 | 0.62 | 1.02 |
| PRIMER0*LETTER3*REMIND0 | VS. | PRIMER1*LETTER3*REMIND1 | 0.79 | 0.61 | 1.01 |
| PRIMER0*LETTER3*REMIND1 | VS. | PRIMER1*LETTER0*REMIND0 | 1.13 | 0.88 | 1.46 |
| PRIMER0*LETTER3*REMIND1 | VS. | PRIMER1*LETTER0*REMIND1 | 1.03 | 0.8 | 1.31 |
| PRIMER0*LETTER3*REMIND1 | VS. | PRIMER1*LETTER1*REMIND0 | 0.93 | 0.73 | 1.18 |
| PRIMER0*LETTER3*REMIND1 | VS. | PRIMER1*LETTER1*REMIND1 | 1.01 | 0.79 | 1.3 |
| PRIMER0*LETTER3*REMIND1 | VS. | PRIMER1*LETTER2*REMIND0 | 0.96 | 0.75 | 1.23 |
| PRIMER0*LETTER3*REMIND1 | VS. | PRIMER1*LETTER3*REMIND0 | 1.03 | 0.81 | 1.32 |
| PRIMER0*LETTER3*REMIND1 | VS. | PRIMER1*LETTER3*REMIND1 | 1.02 | 0.8 | 1.31 |
| PRIMER1*LETTER0*REMIND0 | VS. | PRIMER1*LETTER0*REMIND1 | 0.9 | 0.71 | 1.16 |
| PRIMER1*LETTER0*REMIND0 | VS. | PRIMER1*LETTER1*REMIND0 | 0.82 | 0.64 | 1.04 |
| PRIMER1*LETTER0*REMIND0 | VS. | PRIMER1*LETTER1*REMIND1 | 0.89 | 0.7 | 1.15 |
| PRIMER1*LETTER0*REMIND0 | VS. | PRIMER1*LETTER2*REMIND0 | 0.85 | 0.66 | 1.09 |
| PRIMER1*LETTER0*REMIND0 | VS. | PRIMER1*LETTER3*REMIND0 | 0.91 | 0.71 | 1.17 |
| PRIMER1*LETTER0*REMIND0 | VS. | PRIMER1*LETTER3*REMIND1 | 0.9 | 0.71 | 1.15 |
| PRIMER1*LETTER0*REMIND1 | VS. | PRIMER1*LETTER1*REMIND0 | 0.9 | 0.71 | 1.14 |
| PRIMER1*LETTER0*REMIND1 | VS. | PRIMER1*LETTER1*REMIND1 | 0.99 | 0.77 | 1.26 |
| PRIMER1*LETTER0*REMIND1 | VS. | PRIMER1*LETTER2*REMIND0 | 0.94 | 0.74 | 1.19 |
| PRIMER1*LETTER0*REMIND1 | VS. | PRIMER1*LETTER3*REMIND0 | 1.01 | 0.79 | 1.28 |
| PRIMER1*LETTER0*REMIND1 | VS. | PRIMER1*LETTER3*REMIND1 | 1 | 0.78 | 1.27 |
| PRIMER1*LETTER1*REMIND0 | VS. | PRIMER1*LETTER1*REMIND1 | 1.09 | 0.86 | 1.4 |
| PRIMER1*LETTER1*REMIND0 | VS. | PRIMER1*LETTER2*REMIND0 | 1.04 | 0.82 | 1.32 |
| PRIMER1*LETTER1*REMIND0 | VS. | PRIMER1*LETTER2*REMIND1 | 0.82 | 0.64 | 1.03 |
| PRIMER1*LETTER1*REMIND0 | VS. | PRIMER1*LETTER3*REMIND0 | 1.12 | 0.88 | 1.42 |
| PRIMER1*LETTER1*REMIND0 | VS. | PRIMER1*LETTER3*REMIND1 | 1.1 | 0.87 | 1.4 |
| PRIMER1*LETTER1*REMIND1 | VS. | PRIMER1*LETTER2*REMIND0 | 0.95 | 0.74 | 1.21 |
| PRIMER1*LETTER1*REMIND1 | VS. | PRIMER1*LETTER3*REMIND0 | 1.02 | 0.8 | 1.31 |
| PRIMER1*LETTER1*REMIND1 | VS. | PRIMER1*LETTER3*REMIND1 | 1.01 | 0.79 | 1.29 |
| PRIMER1*LETTER2*REMIND0 | VS. | PRIMER1*LETTER3*REMIND0 | 1.07 | 0.84 | 1.37 |
| PRIMER1*LETTER2*REMIND0 | VS. | PRIMER1*LETTER3*REMIND1 | 1.06 | 0.84 | 1.35 |
| PRIMER1*LETTER3*REMIND0 | VS. | PRIMER1*LETTER3*REMIND1 | 0.99 | 0.78 | 1.26 |
